# Working Memory Deficits in Multiple Sclerosis: An Overview of the Findings

**DOI:** 10.3389/fpsyg.2022.866885

**Published:** 2022-04-28

**Authors:** Zoe Kouvatsou, Elvira Masoura, Vasilios Kimiskidis

**Affiliations:** ^1^Department of Experimental Cognitive Psychology, School of Psychology, Aristotle University of Thessaloniki, Thessaloniki, Greece; ^2^First Department of Neurology, AHEPA Hospital, Aristotle University of Thessaloniki, Thessaloniki, Greece

**Keywords:** working memory, information processing speed, multiple sclerosis, episodic buffer, central executive

## Abstract

Although working memory (WM) and information processing speed (IPS) impairments in multiple sclerosis (MS) have been widely investigated, several questions, regarding the nature of these impairments and their relationship, remain unclear. The aim of this short communication article is to present an overview of our recent research findings regarding (a) the characteristics of WM impairment in MS patients and, more precisely, the degree of impairment observed in each WM’s component, i.e., phonological loop, visuospatial sketchpad, central executive, and episodic buffer and (b) the relationship between IPS and each of the four WM components, in an attempt to expand the existing rather narrow understanding of the interconnection between reduced IPS and WM impairment. Two studies of our research team are presented here and their findings are briefly discussed, highlighting the importance of further research on a specific component, namely the episodic buffer component among MS patients.

## Introduction

Multiple sclerosis (MS) is a chronic, inflammatory, demyelinative, and degenerative disease of the central nervous system. Cognitive decline is a clinically relevant manifestation of MS, mainly involving deficits in information processing speed (IPS; Demaree et al., 1999; DeLuca et al., 2004; Costa et al., 2017) and in working memory (WM; Brassington and Marsh, 1998; Chiaravalloti and DeLuca, 2008; Sandry et al., 2019). Although IPS and WM deficits among patients with MS have attracted significant research interest, crucial research questions about the specific mechanisms that underlie the nature of their impairment and their interactions in MS, remain unanswered.

Our research team attempted to evaluate: (a) the level of impairment in the WM components and (b) the effects of IPS impairments on WM functioning among MS patients, using the theoretical framework of the WM model of Baddeley and Hitch (1974), as modified by Baddeley (2000). According to this WM model (Baddeley and Hitch, 1974; Baddeley, 2000), WM is a mental working place with four components: The phonological loop, the visuospatial sketchpad, the central executive, and the episodic buffer. The phonological loop is the component where verbal information is retained and rehearsed (Baddeley et al., 1984), while the visuo-spatial sketchpad is the component which specializes in constructing and storing spatial and visual information. The episodic buffer integrates information across the WM components and long-term memory, into coordinated sequences of events (episodes; Baddeley, 2000), while the central executive is conceived as a higher order component, which appropriately controls attention and coordinates the other WM subcomponents (Baddeley, 1996). This model for WM has been widely influential and its implications have been tested in both healthy and neurologically impaired participants (Masoura et al., 2021).

### The Degree of Impairment in Each of the Four Working Memory Components

Several studies have been conducted to assess the function of WM in MS patients and the implications of the Baddeley and Hitch’s model have proved to be useful (Thornton et al., 2002; Panou et al., 2009; Fuso et al., 2010; Nathalie et al., 2014). However, these studies have selectively examined only some WM components, while a thorough evaluation of all WM components in an adequate sample of MS patients, was not available yet. Thus, in Kouvatsou et al. (2019), we attempted to investigate in detail the level of impairment in all WM components in a group of patients with MS, using sensitive tasks and assessments for each WM’s component.

Thirty-eight patients diagnosed with MS and 27 matched controls entered the study. All participants were assessed with 12 different cognitive tasks, which measured the four WMs’ components. More specifically, Greek translated and adapted versions of the following tasks were administered: (a) Digit recall, word list recall and non-word list recall, for the assessment of phonological loop (verbal short-term memory). (b) Block recall, mazes recall, and visual patterns recall, to measure visuo spatial sketchpad (visual and spatial short-term memory). (c) Backward digit recall, backward block recall, and listening recall, to assess central executive. (d) Logical memory I, Immediate story recall and Greek verbal learning test, based on the California verbal learning test (Vlahou et al., 2013), and, specifically, the first learning trial (free recall of List A, immediately after hearing the last word of the list) and the free recall of the interference list (List B) were used to assess the episodic buffer component of WM. Participants were instructed, both in List A and B recall, to recall as many words as possible in a random order, immediately after hearing the last word of the list. In order to compare patients’ performance on each variable, taking into account the controls’ performance, raw scores were converted to *z*-scores, using the means and standard deviations of the controls’ group for each variable (Drake et al., 2006; Demery et al., 2010; Hardmeier et al., 2012). Patients’ performance in each variable was dichotomized as non- impaired/impaired performance in relation to controls’ performance, using the cutoff score of two Standard Deviations. Furthermore, repeated measures ANOVA (with *post hoc* Bonferroni comparisons) was used to evaluate the effects between the group (patients-controls) and the four WM components (as dependent variables). Results revealed that patients’ performance was significantly impaired, in comparison to controls’, in all the WM tasks, expect from the visual patterns tasks. The visual subcomponent of visuospatial sketchpad was found to be preserved, as the scores of MS patients in the visual patterns task did not present significant differences from controls. Moreover, it was revealed that the episodic buffer was more heavily affected than the phonological loop, the central executive and the spatial subcomponent of visuospatial sketchpad, which were found to be equally impaired. Given that the episodic buffer is the least investigated system so far, these results are important, since they emphasize the role of the binding process in cognitive tasks and the specific impairment among MS patients.

### Information Processing Speed and Working Memory Relationship in Multiple Sclerosis

With regard to the relationship of IPS and WM, there are several studies suggesting that the primary deficit in MS patients is IPS, affecting primarily the encoding of information, while impairment in WM is considered more as a result of reduced IPS (DeLuca et al., 2004; Lengenfelder et al., 2006; Costa et al., 2017). On the other side, other studies have suggested that deficits in WM and IPS are developed separately, affecting independently the encoding processes (D’Esposito et al., 1996; Landrø et al., 2004). Our research team evaluated the effect of IPS on the four WM components function, i.e., phonological loop, visuospatial sketchpad, central executive and episodic buffer (Kouvatsou et al., 2020). Different tasks were administered to 38 patients with MS to evaluate each WM component: word lists recall for the phonological loop, block recall for the spatial component of visuospatial sketchpad, listening recall for the central executive and immediate story recall, for the episodic buffer function. The symbol digit modalities test -in its oral form- was administered to evaluate IPS. Statistical analysis was performed using Pearson’s correlation coefficient (Pearson’s *r*) to assess the relationship of clinical, psycho-affective, and cognitive variables, while a within- subjects analysis was designed using hierarchical regression analyses in order to evaluate the influence of IPS on the related WM components.

Results showed that IPS was significantly related only to the central executive and episodic buffer components, while impairments in the two simple storage devices (phonological loop and visuospatial sketchpad) were found to operate more independently. However, when chronological age was inserted in the regression model, only the episodic buffer’s capacity was significantly predicted by IPS. We interpreted these findings as an indication that IPS and WM in patients with MS have a partially interdependent relationship.

## Discussion

We conducted two different studies in order to evaluate the nature of the WM impairment and the relationship of IPS and WM in MS patients. The theoretical framework of the WM Model of Baddeley and Hitch (1974) and Baddeley (2000), in accordance with previous studies (Thornton et al., 2002; Panou et al., 2009; Fuso et al., 2010; Nathalie et al., 2014) was used to interpret our findings.

With regard to the nature of WM impairment, we estimated the degree of impairment in each of the four WM components (Kouvatsou et al., 2019). Results revealed that WM components are differentially affected in patients with MS and, more precisely, impairment in the episodic buffer was found to be more profound than impairment in the phonological loop, central executive and the spatial subcomponent of visuospatial sketchpad. Another interesting finding was that no impairment was noticed in the visual subcomponent of visuospatial sketchpad.

Findings were discussed in the context of the existing knowledge regarding WM impairment in MS. According to Baddeley et al. (2010), the episodic buffer is the component used to store information in the form of episodes, i.e., information integrated into a single complex structure, while the central executive is a component involved in more active processes of information binding. Our results showed that, although impairments in both creating and holding episodes in WM are significant in MS patients, it seems that a more profound deficit is noticed in the process of storing episodes. Moreover, our results suggested that only the visual component of visuospatial sketchpad remained intact among MS patients, while the spatial component was found to be significantly impaired and we highlighted that this observed differential impact on the visual and spatial features of the visuospatial sketchpad in MS should be further explored.

With regard to the relationship between IPS and WM, we evaluated the relationship of IPS with the four WM components, i.e., phonological loop, visuospatial sketchpad, central executive, and episodic buffer (Kouvatsou et al., 2020). Results revealed that the complex storage WM component, i.e., central executive and episodic buffer, are more closely related to IPS, than the simple storage components, i.e., phonological loop and visuospatial sketchpad. However, the role of the executive component of WM, namely the central executive, in IPS impairment was found to be insignificant when chronological age was included in the analysis. Thus, it was suggested that central executive may not be directly affected by impaired IPS in patients with MS, while other general factors, such as chronological age, may mediate their relationship. In other words, IPS was found to have a more direct effect on the episodic buffer function than the central executive.

Overall, the findings of our studies provide further insight on the function of WM components and their relationship with IPS in patients with MS. Furthermore, these findings might be useful in developing appropriate cognitive rehabilitation programs, as emerging evidence suggests that there are certain aspects of WM that could be targeted and trained to ameliorate the encoding, consolidation, and retrieval of information in MS patients. Future research should further clarify the relationship between the four WM components, the IPS and the role of mediating factors, such as age, education, disease burden, and visual functions.

## Author Contributions

ZK, EM, and VK contributed to the conception and design of the review. ZK and EM wrote the first draft of the manuscript. All authors contributed to the revision of the first draft and approved the submitted version.

## Conflict of Interest

The authors declare that the research was conducted in the absence of any commercial or financial relationships that could be construed as a potential conflict of interest.

## Publisher’s Note

All claims expressed in this article are solely those of the authors and do not necessarily represent those of their affiliated organizations, or those of the publisher, the editors and the reviewers. Any product that may be evaluated in this article, or claim that may be made by its manufacturer, is not guaranteed or endorsed by the publisher.
